# Hepatitis B Virus-Specific miRNAs and Argonaute2 Play a Role in the Viral Life Cycle

**DOI:** 10.1371/journal.pone.0047490

**Published:** 2012-10-16

**Authors:** C. Nelson Hayes, Sakura Akamatsu, Masataka Tsuge, Daiki Miki, Rie Akiyama, Hiromi Abe, Hidenori Ochi, Nobuhiko Hiraga, Michio Imamura, Shoichi Takahashi, Hiroshi Aikata, Tomokazu Kawaoka, Yoshiiku Kawakami, Waka Ohishi, Kazuaki Chayama

**Affiliations:** 1 Department of Gastroenterology and Metabolism, Applied Life Sciences, Institute of Biomedical & Health Sciences, Hiroshima University, Hiroshima, Japan; 2 Laboratory for Digestive Diseases, Center for Genomic Medicine, RIKEN, Hiroshima, Japan; 3 Liver Research Project Center, Hiroshima University, Hiroshima, Japan; 4 Natural Science Center for Basic Research and Development, Hiroshima University, Hiroshima, Japan; 5 Department of Clinical Studies, Radiation Effects Research Foundation, Hiroshima, Japan; Yonsei University College of Medicine, Republic of Korea

## Abstract

Disease-specific serum miRNA profiles may serve as biomarkers and might reveal potential new avenues for therapy. An HBV-specific serum miRNA profile associated with HBV surface antigen (HBsAg) particles has recently been reported, and AGO2 and miRNAs have been shown to be stably associated with HBsAg in serum. We identified HBV-associated serum miRNAs using the Toray 3D array system in 10 healthy controls and 10 patients with chronic hepatitis B virus (HBV) infection. 19 selected miRNAs were then measured by quantitative RT-PCR in 248 chronic HBV patients and 22 healthy controls. MiRNA expression in serum versus liver tissue was also compared using biopsy samples. To examine the role of AGO2 during the HBV life cycle, we analyzed intracellular co-localization of AGO2 and HBV core (HBcAg) and surface (HBsAg) antigens using immunocytochemistry and proximity ligation assays in stably transfected HepG2 cells. The effect of AGO2 ablation on viral replication was assessed using siRNA. Several miRNAs, including miR-122, miR-22, and miR-99a, were up-regulated at least 1.5 fold (P<2E-08) in serum of HBV-infected patients. AGO2 and HBcAg were found to physically interact and co-localize in the ER and other subcellular compartments. HBs was also found to co-localize with AGO2 and was detected in multiple subcellular compartments. Conversely, HBx localized non-specifically in the nucleus and cytoplasm, and no interaction between AGO2 and HBx was detected. SiRNA ablation of AGO2 suppressed production of HBV DNA and HBs antigen in the supernatant.

**Conclusion:**

These results suggest that AGO2 and HBV-specific miRNAs might play a role in the HBV life cycle.

## Introduction

Hepatitis B virus (HBV) is a partially double-stranded DNA virus in the Hepadnaviridae family [1]. New therapies are urgently needed for the 350 million chronically infected individuals who face a significantly elevated lifetime risk of cirrhosis and hepatocellular carcinoma [2], [3]. Recent insight into the role of non-coding RNAs in the liver has highlighted potential applications of microRNAs (miRNAs) in HBV diagnosis and treatment [4], [5], [6], [7], [8], [9].

MiRNAs are a class of short non-coding RNAs involved in post-transcriptional gene regulation of multiple pathways [10]. In contrast to messenger RNAs, exosome-free extracellular miRNAs may be nuclease-resistant and remain in circulation for long periods of time by being stably bound to AGO2, a component of the RNA-induced silencing complex [11]. The origin and function of these extracellular miRNAs is unclear, but they may serve as biomarkers for liver injury and cancer [4]. Elucidating the function of hepatic miRNAs in HBV infection is important in the development of strategies to eradicate the virus and assess the risk of HCC. A number of miRNAs have been shown to be up- or down-regulated in HBV infection [4], [12], [13]. Noting that the defective hepatitis delta virus co-opts HBsAg subviral particles for export, Novellino et al. hypothesized that HBsAg subviral particles might also sequester miRNAs from the liver [5]. Using HBsAg immunoprecipitation, they identified a set of liver-specific and immune regulatory AGO2-bound miRNAs associated with HBsAg.

These reports suggest that AGO2 and a specific subset of miRNAs may participate in HBV replication, either as part of a host anti-HBV defense or as viral strategy to exploit or evade the RISC machinery. In this study, we examined serum miRNA expression in chronic HBV and healthy individuals and found a specific subset of miRNAs that are over-expressed in HBV-positive patients and in which miR-122 was strongly up-regulated. To determine whether components of the miRNA system are associated with other HBV components, we performed subcellular localization experiments with viral proteins and AGO2.

## Materials and Methods

### Study Subjects

We performed a series of experiments to compare miRNA profiles of healthy and HBV-infected individuals in serum and liver tissue. All patients had chronic hepatitis B and agreed to provide blood samples for a viral hepatitis study. Patient profiles are shown in Table 1. Histopathological diagnosis was made according to the criteria of Desmet et al. [14]. The study protocol conforms to the ethical guidelines of the 1975 Declaration of Helsinki, and all patients provided written informed consent. This study was approved a priori by the ethical committee of Hiroshima University.

**Table 1:** Clinical characteristics of chronic hepatitis B virus patients (n = 248).

Factor	Value
Age	44 (15–76)
Sex (male/female)	169/77
Alanine aminotransferase (IU/l)	56 (10–1867)
Aspartate aminotransferase (IU/l)	43.5 (15–982)
HBV DNA (IU/ml)	6.3 (1.8–9.1)
Liver fibrosis (1/2/3/4)	69/102/46/26
Necroinflammatory activity (0/1/2/3/4)	1/70/127/45/0
γ-glutamyl transpeptidase (IU/l)	43 (9–459)
Alpha-fetoprotein (µg/l)	6.15 (0–9400)
Promthrombin time (s)	93 (0–146)
Albumin (g/dl)	4.4 (0–5.2)
Platelets (x10^4^/mm^3^)	16.75 (1–36)
HBsAg (IU/l)	2765 (0.05–239000)
HBeAg (−/+)	115/127
HBeAb (−/+)	113/128

Continuous variables are shown as median and range, and categorical variables are shown as counts.

Fibrosis and necroinflammatory activity were scored according to the criteria of Desmet et al. [14].

### miRNA Expression Levels in Serum

miRNA expression in serum samples was measured using the Toray Industries miRNA analysis system, in which serum miRNA samples were hybridized to 3D-Gene human miRNA ver12.1 chips containing 900 miRNAs (Toray Industries, Inc., Tokyo, Japan). MiRNA gene expression data were scaled by global normalization, and differential expression was analyzed using the limma package in the R statistical framework. Serum was collected from 20 patients with high HBV DNA and HBsAg levels and with either high (>42 IU/l) or low (≤42 IU/l) ALT levels. Serum from the 10 low ALT patients was analyzed as a mixture, whereas serum from each of the 10 high ALT patients was analyzed both separately and as a mixture. For comparison with healthy controls we collected separate mixtures of serum from 10 healthy females and 12 healthy males. Serum samples from each healthy female were also measured separately. All healthy controls were negative for HBsAg, HBcAb, and HCV Ab. For comparison with miRNA expression in hepatocytes, miRNA expression was measured in non-tumor biopsy tissue from an HBV-infected patient and compared to non-cancerous liver tissue samples from two patients without HBV or HCV infection.

### Quantitative Real-time Polymerase Chain Reaction miRNA Analysis

Using real-time polymerase chain reaction (RT-PCR) we measured the expression of 19 miRNAs in serum from 248 patients with chronic HBV infection and from 10 healthy females and 12 healthy males. Circulating microRNA was extracted from 300 µl of serum samples using the mirVana PARIS Kit (Ambion, Austin, TX) according to the manufacturer’s instructions. RNA was eluted in 80 µl of nuclease free water and reverse transcribed using TaqMan MicroRNA Reverse Transcription Kit (Life Technologies Japan, Tokyo, Japan). *Caenorhabditis elegans* miR-238 (cel-miR-238) was spiked to each sample as a control for extraction and amplification steps. The reaction mixture contained 5 µl of RNA solution, 2 µl of 10× reverse transcription buffer, 0.2 µl of 100 mM dNTP mixture, 4 µl of 5× RT primer, 0.25 µl of RNase inhibitor and 7.22 µl of nuclease free water in a total volume of 20 µl. The reaction was performed at 16°C for 30 min followed by 42°C for 30 min. The reaction was terminated by heating the solution at 85°C for 5 min. MiRNAs were amplified using primers and probes provided by Applied Biosystems using TaqMan MicroRNA assays according to the manufacturer’s instructions. The reaction mixture contained 12.5 µl of 2× Universal PCR Master Mix, 1.25 µl of 20× TaqMan Assay solution, 1 µl of reverse transcription product and 10.25 µl of nuclease free water in a total volume of 25 µl. Amplification conditions were 95°C for 10 min followed by 50 denaturing cycles for 15 sec at 95°C and annealing and extension for 60 sec at 60°C in an ABI7300 thermal cycler. For the cel-miR-238 assay, a dilution series using chemically synthesized miRNA was used to generate a standard curve that permitted absolute quantification of molecules.

### Pathway Analysis

Target genes of differentially expressed miRNAs were predicted based on agreement among three miRNA prediction tools, miRanda, miRBase, and TargetScan. Gene Set Enrichment Analysis (http://www.broadinstitute.org/gsea) was used to identify significantly over-represented gene ontology (GO) terms among the predicted targets.

### Plasmid Construction

The construction of wild-type HBV 1.4 genome length, pTRE-HB-wt, was described previously [15]. We used pTRE2 vector without pTet-off vector and doxycycline because a sufficient amount of HBV transcript was produced from internal HBV promoters, and transcription from the pTRE2 promoter is negligible under these conditions. The nucleotide sequence of the HBV genome that we cloned into plasmids pTRE-HB-wt was deposited into GenBank under accession number AB206817.

### Cell Culture

HepG2 cells, derived from a human hepatoma cell line, were grown in Dulbecco’s modified Eagle’s medium (DMEM) supplemented with 10% (v/v) fetal bovine serum at 37°C and under 5% CO_2_. For the production of stably transfected cell lines, HepG2 cells were transfected with 20µg of the plasmid pTRE-HB-wt by calcium precipitation and the transfected cells were selected with 400µg/ml hygromycin-included DMEM. Sixty colonies were isolated, and clones that were positive for both HBs and HBe antigens were selected. Finally, one cell line named T23 was selected and used for further experiments. T23 cells continuously produced more than 6 log copies/ml of HBV DNA in supernatant over more than 12 months (data not shown).

### Immunocytochemistry

Co-localization between AGO2 and several HBV proteins (HBc, HBs, and HBx) was analyzed using immunocytochemistry, followed by cellular localization assays using antibodies targeting various sub-cellular compartments. HepG2 or T23 cells were seeded in 2-well chamber plates and harvested 48 hours after seeding. The cells were washed with PBS and fixed with 4% (v/v) paraformaldehyde. After fixation, the cells were stained with several primary antibodies (Table S1). The bound antibodies were detected with an Alexa 488-conjugated antibody against rabbit IgG (1∶2000) or Alexa 568-conjugated antibody against mouse IgG (1∶2000), respectively (Molecular Probes, Eugene, OR). Nuclei were counterstained with 6-diamidino-2-phenylindole (DAPI) (Vector laboratories, Burlingame, CA). The stained cells were examined with a Fluoview FV10i microscope (Olympus, Tokyo, Japan).

### In situ Proximity Ligation Assay

We used proximity ligation assays (PLA) to determine whether AGO2 and HBc physically interact. PLA is a recent method to detect protein-protein interactions using protein-DNA conjugates that can be detected using fluorescence microscopy [16]. PLA improves on traditional immunoassays by directly detecting even weak or transient protein interactions [16]. HepG2 and T23 cells were seeded in 2-well chamber plates and harvested 48 hours after seeding. The cells were washed with PBS and fixed with 4% (v/v) paraformaldehyde. After fixation, the cells were stained with primary antibodies. The primary antibodies used are listed in Table S1. After overnight incubation with primary antibody at 4°C, PLA was performed using Duolink II PLA probe anti-rabbit plus and anti-mouse minus and Duolink II Detection Reagents Orange (Olink, Uppsala, Sweden) following the manufacturer’s protocol. Nuclei were counterstained with DAPI. Imaging was performed using a Fluoview FV10i microscope.

### Analysis of Supernatant HBV Production by RNA Interference Against AGO2

To investigate the necessity of AGO2 for HBV production, we performed RNA interference assay using T23 cells that are HepG2 cells stably transfected with the plasmid pTRE-HB-wt. We used Silencer Select Pre-designed siRNA small interfering RNA targeting *AGO2* (#s25932, Ambion, Austin, TX) and Silencer Select Negative Control #1 siRNA for control (Ambion). T23 cells were transfected with one of the siRNA oligonucleotides (10 nM) using Lipofectamine RNAiMAX (Invitrogen, Carlsbad, CA) according to the manufacturer’s instructions. To examine the knockdown effect of siRNAs against *AGO2* by real-time quantitative RT-PCR, T23 cells transfected with siRNAs were harvested 72 hours after transfection. Total RNA was isolated using the QuickGene RNA cultured cell kit S (Fujifilm, Tokyo, Japan). One µg of each RNA sample was reverse transcribed with the SuperScript VILO cDNA Synthesis kit (Invitrogen). First-strand complementary DNA (cDNA) was amplified with specific primers for the coding sequence of *AGO2*. The primers were as follows: forward, 5′-CCAGCATACTACGCTCACCT-3′; reverse, 5′-CAGAGTGTCTTGGTGAACCTG-3′. We quantified *AGO2* mRNA with EXPRESS SYBR Green ER qPCR Supermix Universal (Invitrogen) according to the manufacturer’s instructions. Amplification and detection were performed using the Mx3000P Multiplex quantitative PCR system (Stratagene, La Jolla, CA). Results were normalized to the transcript levels of the housekeeping reference gene glyceraldehyde-3-phosphate dehydrogenase (*GAPDH*). Three to seven days after transfection, the culture media were collected to examine HBV production in supernatant. HBs antigen was measured quantitatively using the Abbott chemiluminescence immunoassay kit (Abbott Japan, Tokyo, Japan). HBV DNA levels were determined by Cobas TaqMan HBV standardized real-time PCR assay (Roche Molecular Systems, Pleasanton, CA). Results are expressed in log10 international units/ml. We also evaluated viability of cells using the Cell Counting kit-8 (Dojindo Laboratories, Kumamoto, Japan) at 3, 5 and 7 days after transfection, according to the manufacturer’s instructions. All assays were performed in triplicate, and the results are expressed as mean ± SD.

### Statistical Analysis

All analyses were performed using the R statistical package (http://www.r-project.org). Continuous variables are reported using the median and range. Moderated t statistics or Mann Whitney U tests were used to detect significant associations, as appropriate, and P-values were adjusted for multiple testing based on the false discovery rate.

## Results

### MiRNA Microarray Results

We performed miRNA microarray analysis to identify HBV-associated differences in serum miRNA profiles between 10 chronic HBV patients and 10 healthy controls (Fig. S1). 26 miRNAs with an absolute log fold change greater than 1.5 were found to be significantly (P_FDR_ <0.05) up-regulated in serum of HBV patients, and 8 miRNAs were significantly down-regulated (Table 2). MiR-122, miR-22, and miR-99a levels were the most strongly up-regulated in serum of HBV-infected patients, and levels of miR-575, miR-125a-3p, and miR-4294 were the most down-regulated. We also examined miRNAs associated with presence of HBe antigen or HBe antibody, but no miRNAs were significant following correction for multiple testing (data not shown).

**Table 2:** Top 10 up- or down-regulated serum miRNAs associated with chronic HBV infection.

Sample	Direction	miRNA	logFC	AveExpr	t	P	P_FDR_
Serum	Up	hsa-miR-122	5.97	9.09	12.84	3.27E−12	3.06E−09
		hsa-miR-99a	2.59	6.20	10.73	2.11E−10	2.19E−08
		hsa-miR-22	2.49	9.55	10.47	2.10E−10	2.19E−08
		hsa-miR-191	2.19	8.42	11.87	1.68E−11	3.93E−09
		hsa-miR-642b	2.03	10.07	9.93	5.92E−10	4.26E−08
		hsa-miR-125b	1.95	5.99	8.72	9.91E−09	4.21E−07
		hsa-miR-486-3p	1.79	9.09	8.01	3.19E−08	9.95E−07
		hsa-miR-378	1.78	5.97	9.94	9.00E−10	6.02E−08
		hsa-miR-320d	1.70	7.19	7.88	4.25E−08	1.21E−06
		hsa-miR-23b	1.69	8.99	7.62	7.64E−08	1.93E−06
	Down	hsa-miR-575	−2.10	8.35	−10.00	5.20E−10	4.05E−08
		hsa-miR-125a-3p	−1.99	7.22	−11.91	1.56E−11	3.93E−09
		hsa-miR-4294	−1.75	11.82	−11.37	4.07E−11	7.63E−09
		hsa-miR-92a-2*	−1.64	11.03	−7.70	6.36E−08	1.75E−06
		hsa-miR-1202	−1.59	8.60	−12.41	6.72E−12	3.14E−09
		hsa-miR-30c-1*	−1.31	6.29	−8.66	1.12E−08	4.35E−07
		hsa-miR-1275	−1.19	9.91	−7.50	1.00E−07	2.35E−06
		hsa-miR-3197	−1.05	11.46	−8.58	9.24E−09	4.21E−07
		hsa-miR-1908	−1.03	13.75	−9.05	3.49E−09	2.04E−07
Mixture	Up	hsa-miR-122	6.80	9.09	20.51	1.09E−06	0.001
		hsa-miR-99a	2.58	6.34	9.32	9.80E−05	0.037
		hsa-miR-22	2.07	8.60	3.16	0.020	0.528
		hsa-miR-125b	2.03	6.29	5.09	0.002	0.264
		hsa-miR-1915*	1.80	8.32	6.24	0.001	0.158
		hsa-miR-3648	1.69	14.16	5.06	0.002	0.264
		hsa-miR-642b	1.64	9.82	4.49	0.004	0.377
		hsa-miR-1288	1.39	6.43	3.56	0.012	0.528
		hsa-miR-325	1.30	4.91	2.87	0.047	0.586
		hsa-miR-486-3p	1.29	8.98	3.87	0.009	0.480
	Down	hsa-miR-575	−1.95	8.43	−6.38	0.001	0.158
		hsa-miR-4294	−1.79	11.95	−5.99	0.001	0.158
		hsa-miR-654-3p	−1.35	5.36	−2.99	0.042	0.569
		hsa-miR-1202	−1.24	8.52	−3.97	0.008	0.480
		hsa-miR-1237	−1.06	7.52	−3.10	0.022	0.531
		hsa-miR-744	−1.03	9.51	−2.91	0.028	0.545

Expression levels were compared using moderated t-statistics, and P-values were corrected for multiple testing using the false discovery rate.

logFC: log2 fold-change between patients with chronic HBV infection relative to healthy individuals.

AveExpr: The average log2 expression level for each miRNA over all samples.

t: moderated t-statistic for patients with chronic HBV infection compared to healthy individuals P for each miRNA.

P: uncorrected P-value for t-test.

P_FDR_: P-value adjusted for multiple testing based on the false discovery rate.

### Analysis of Serum Sample Mixtures from HBV-infected Patients and Healthy Controls

In addition to individual serum samples, we also examined 4 pooled serum samples as follows: 10 healthy males, 10 healthy females, 10 HBV patients with low ALT levels, and 10 HBV patients with high ALT levels (Fig. S2). In agreement with results from individual analysis, miR-122 and miR-99 levels were significantly higher in serum from HBV serum samples compared to healthy control samples (Table 2). Corresponding results with a log change greater than 1.5 were found for several other miRNAs, including miR-22, miR-642b, miR-125b (up-regulated) and miR-575 and miR-4294 (down-regulated), but results were not significant following correction for multiple testing in the mixture samples due to the small number of samples compared.

### RT-PCR Analysis

Serum levels of 19 miRNAs were analyzed using quantitative RT-PCR analysis of 250 chronic HBV patients and 20 healthy controls. Several miRNAs (miR-122, miR-22, miR-99a, miR-720, miR-125b, and miR-1275) were significantly up-regulated in serum from HBV-infected patients (Table 3). Agreement of microarray and RT-PCR results was strongest for up-regulation of miR-122, miR-22, and miR-125b in serum of HBV patients. To determine whether there is a linear relationship between HBV markers and HBV-associated miRNAs, we analyzed the correlation between HBsAg and 6 up-regulated miRNAs. MiR-122, miR-99a, and miR-125b levels were found to be significantly correlated with HBsAg levels with R^2^>0.5 (Fig. S3). These three miRNAs were also significantly correlated with HBV DNA titers, with R^2^ of about 0.4 (Fig. S4). MiR-122 and miR-22 were significantly but diffusely associated with serum ALT levels (R^2^>0.2; Fig. S5). To identify miRNAs associated with different phases of HBV infection, we also analyzed the 6 significantly up-regulated miRNAs with respect to the presence of HBe antigen and antibody. MiR-122, miR-99a, miR-720, and miR-125b were each highly significantly elevated in chronic HBV patients who were positive for the HBe antigen (P<4.0E−07; Fig. S6). Similarly, each miRNA was significantly elevated in chronic HBV patients who were negative for the HBe antibody (P<9.1E−05; Fig. S7).

**Figure 1:**
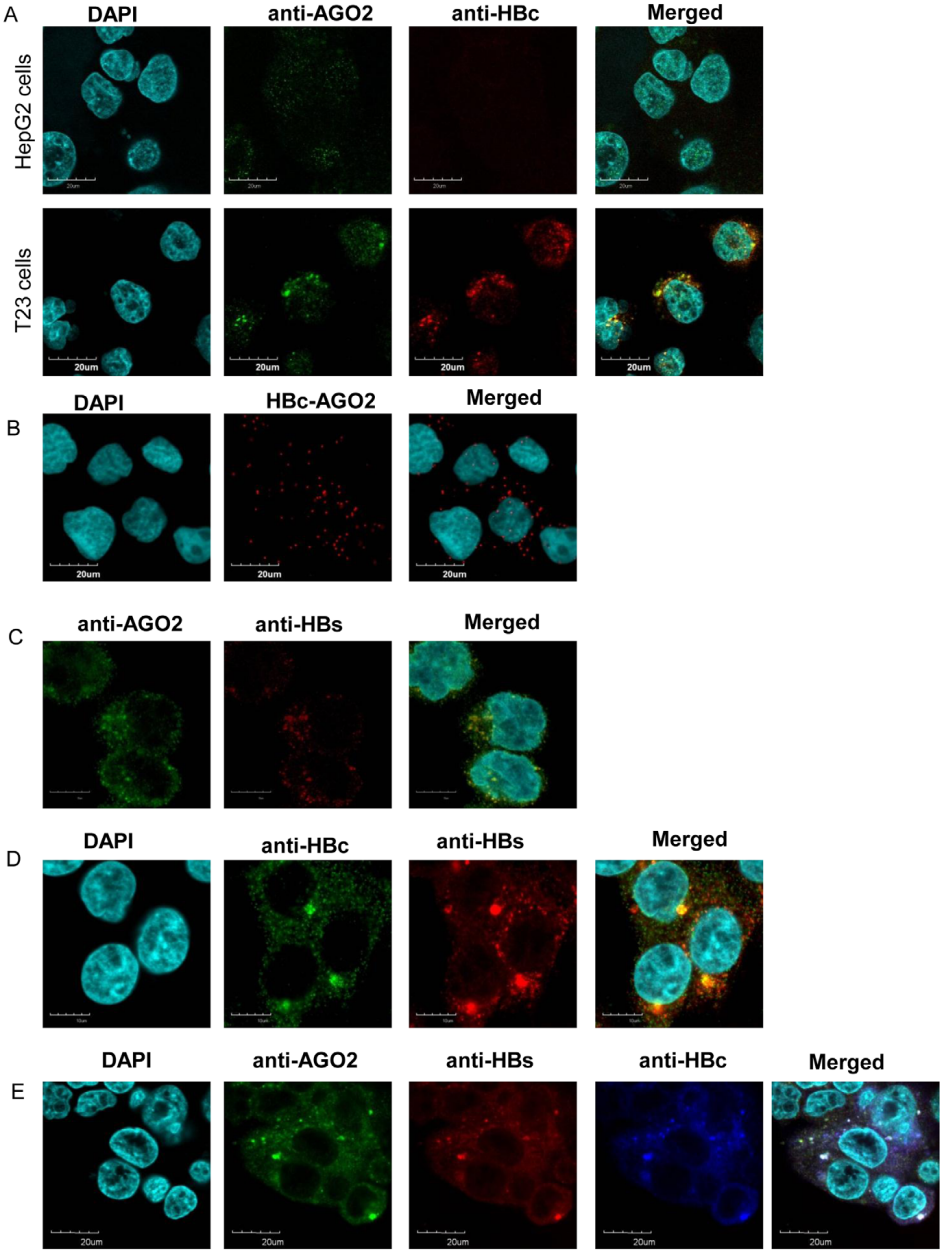
Co-localization of HBcAg and HBsAg with AGO2 in stably transfected T23 cells. A) Anti-AGO2 and anti-HBc staining overlapped in stably transfected T23 cells, but not in HepG2 control cells, suggesting an interaction between HBc and AGO2. B) HBc-AGO2 was detected in T23 but not HepG2 cells using proximity ligation assays (PLA), suggesting a protein-protein interaction between HBcAg and AGO2. C) Overlap of anti-AGO2 and anti-HBs staining suggests co-localization of HBs and AGO2. D) Anti- HBc, and anti-HBs staining overlapped in T23 cells, which may indicate that HBc and HBs co-localize. E) Overlap of anti-AGO2, anti-HBc, and anti-HBs staining in T23 cells suggests that all three proteins may co-localize.

**Table 3:** Quantitative RT-PCR results of selected miRNAs associated in serum of chronic HBV patients.

Factor	Total (n = 270)	HBV (n = 248)	Healthy (n = 22)	P
hsa-miR-122/cel-miR-238	0.1513 (0.0068–2.5)	0.1635 (0.0068–2.5)	0.02074 (0.013–0.04)	1.19E−13
hsa-miR-22/cel-miR-238	0.3 (0.06–1.7)	0.3028 (0.06–1.7)	0.2252 (0.11–0.48)	6.35E−03
hsa-miR-99a/cel-miR-238	0.09121 (0.0046–2.4)	0.102 (0.0086–2.4)	0.0136 (0.0046–0.051)	4.61E−12
hsa-miR-720/cel-miR-238	0.1206 (0.024–3.7)	0.1345 (0.031–3.7)	0.04274 (0.024–0.12)	8.93E−11
hsa-miR-125b/cel-miR-238	0.09732 (0.0066–3.1)	0.1131 (0.0066–3.1)	0.02255 (0.0066–0.05)	1.92E−11
hsa-miR-1275/cel-miR-238	0.4842 (0.099–1.6)	0.5046 (0.099–1.6)	0.4044 (0.24–0.6)	0.010781066
hsa-miR-1826/cel-miR-238	0.5023 (0.14–4.6)	0.5583 (0.26–4.6)	0.33 (0.14–1.4)	7.23E−03
hsa-miR-1308/cel-miR-238	2.831 (1.1–6.9)	2.578 (1.1–6.9)	3.113 (2.3–4.7)	0.223164946
hsa-miR-923/cel-miR-238	3.8 (1.8–9.6)	4.141 (1.8–9.6)	3.01 (2–5)	0.104331611
hsa-miR-1280/cel-miR-238	1.089 (0.36–5)	1.332 (0.6–5)	0.5275 (0.36–0.8)	1.06E−05
hsa-miR-26a/cel-miR-238	1.221 (0.34–3.4)	1.221 (0.34–3.4)	1.231 (0.82–2.4)	0.532171224
hsa-let-7a/cel-miR-238	0.9608 (0.2–2.5)	0.9211 (0.2–2.5)	1.074 (0.71–1.9)	0.235258945
hsa-let-7f/cel-miR-238	1.134 (0.052–2.6)	1.126 (0.052–2.6)	1.143 (0.8–1.7)	0.639411853
hsa-let-7d/cel-miR-238	1.147 (0.35–1.9)	1.106 (0.35–1.8)	1.231 (0.73–1.9)	2.88E−01
hsa-miR-638/cel-miR-238	1.23 (0.3–7)	1.082 (0.3–7)	1.366 (0.68–4)	0.288244047
hsa-miR-1908/cel-miR-238	1.369 (0.45–3.2)	1.357 (0.45–1.9)	1.447 (0.7–3.2)	0.370765019
hsa-miR-34a/cel-miR-238	0.07502 (0.013–1.2)	0.108 (0.026–1.2)	0.02738 (0.013–0.044)	1.41E−05
hsa-miR-886-5p/cel-miR-238	1.627 (0.54–3.6)	1.773 (0.54–3.6)	1.55 (0.97–2.7)	0.478520977

Expression levels were compared using the Mann-Whitney U test.

**Figure 2:**
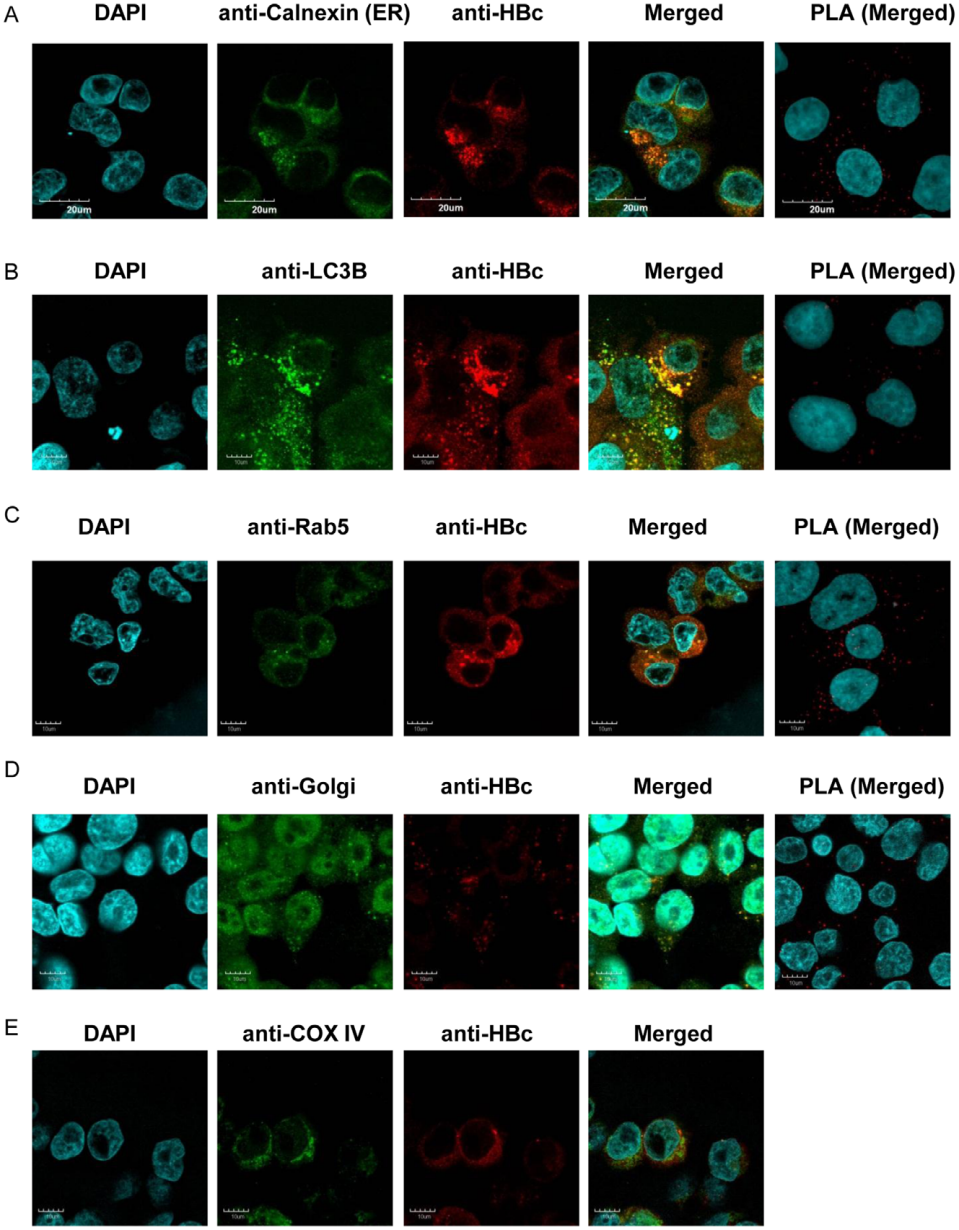
Interactions between HBc and HBs. A) Co-localization of anti-HBc and anti-Calnexin staining by immunocytochemistry and PLA analysis indicate that HBc probably localizes in the ER. Overlap with B) anti-LC3B, C) anti-Rab5, and D) anti-Golgi staining suggests that HBc probably also localizes in autophagosomes, endosomes, and Golgi, respectively. E) However, no overlap was observed with anti-COX IV staining, indicating that HBc probably does not localize at mitochondria.

**Figure 3:**
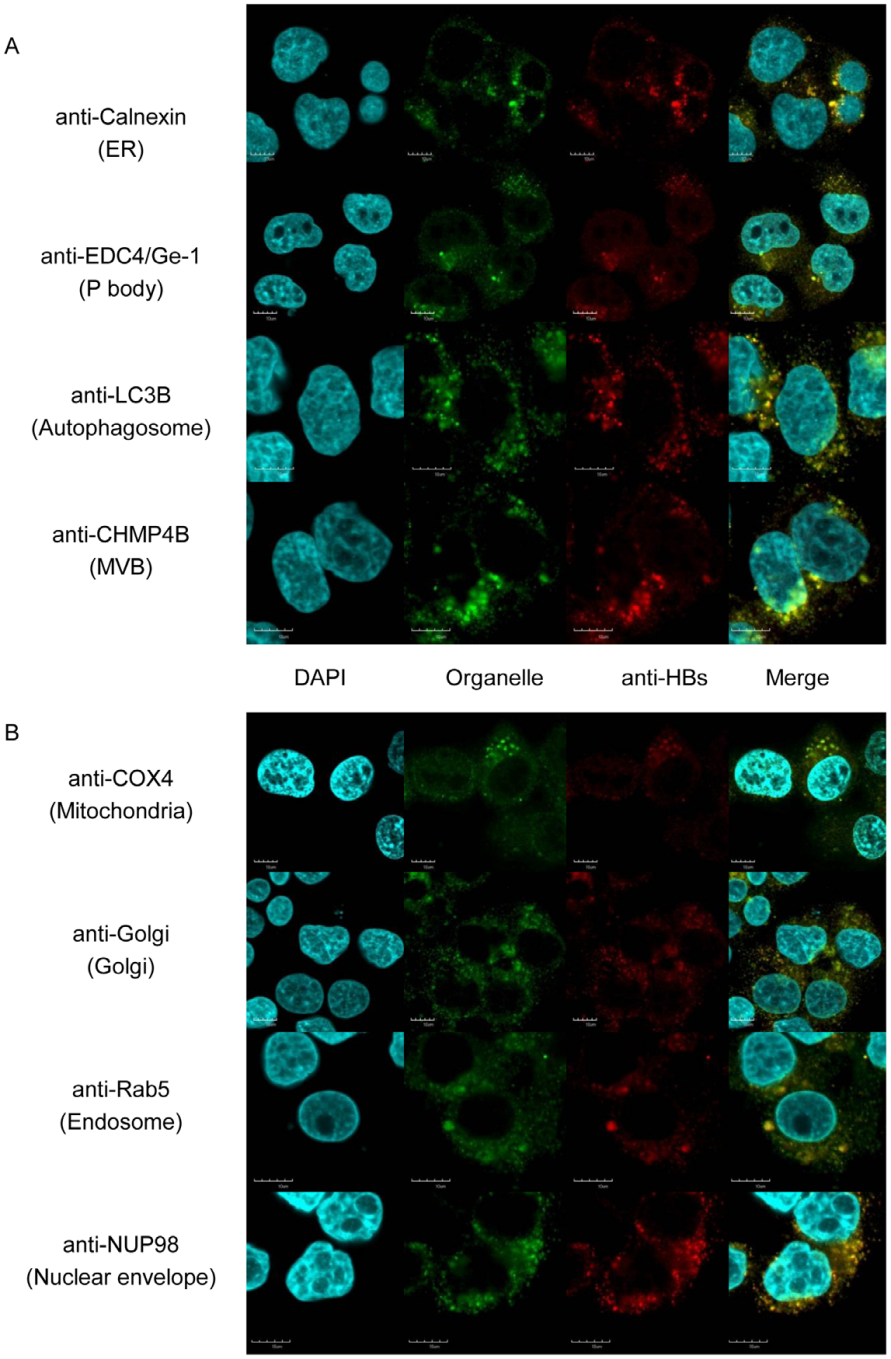
HBsAg localization. A) Co-localization of anti-HBs suggests that HBs localizes in the ER, processing bodies, autophagosomes, and multivesicular bodies, B) and more diffusely in mitochondria, Golgi, endosomes, and at the nuclear envelope.

**Figure 4:**
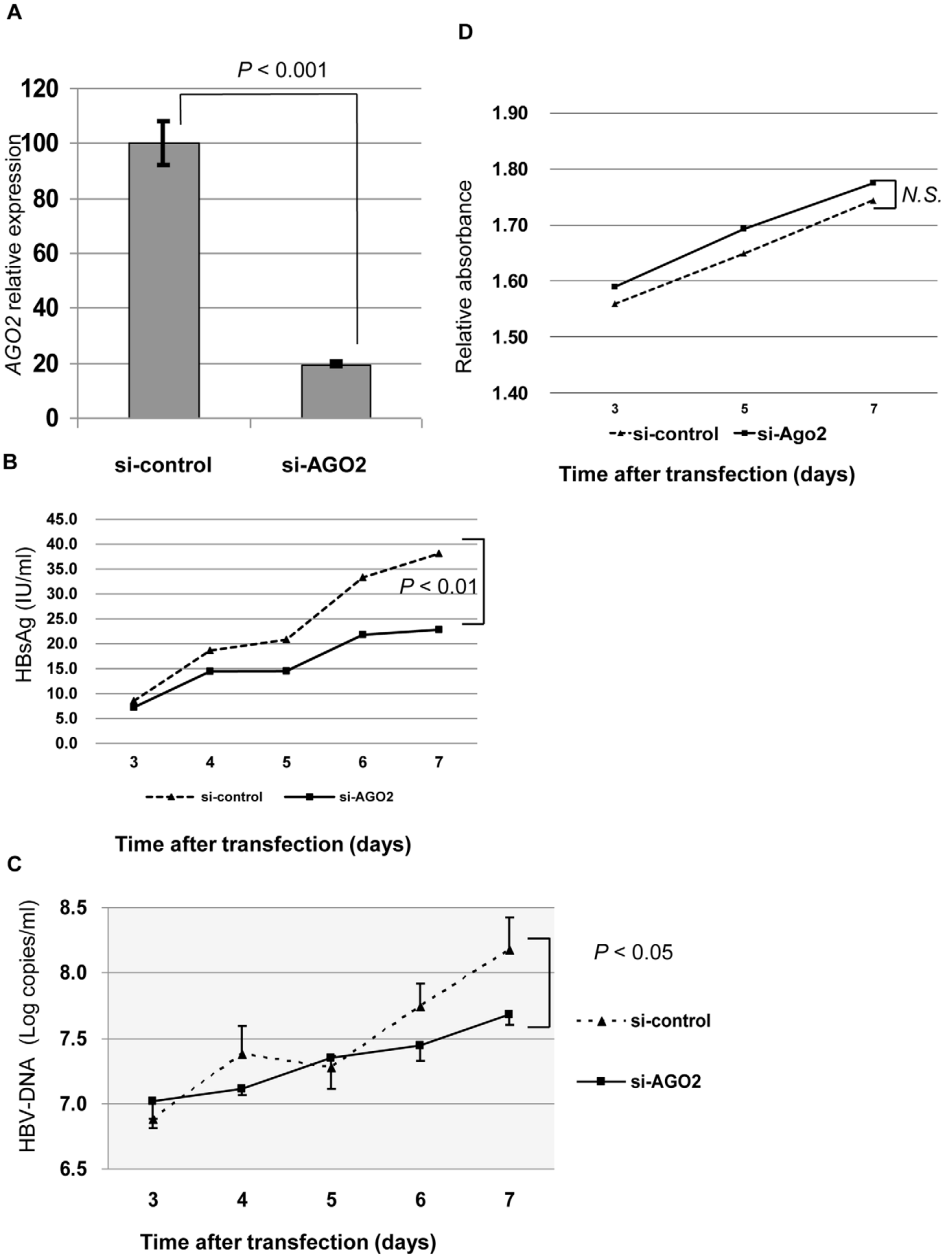
siRNA knock down of AGO2 expression. A) Knock down of *AGO2* expression in T23 cells by specific siRNAs for *AGO2* or control siRNAs, confirmed by real-time quantitative RT-PCR analysis. B) Supernatant HBs antigen, and C) HBV-DNA were measured. Both were higher in supernatant of cells transfected with si-control than in cells transfected with si-*AGO2*. D) There was no significant difference in cell viability between cells transfected with si-control compared to those with si-*AGO2*.

### Pathway Analysis

Predicted gene targets of up-regulated miRNAs were most strongly associated with the GO term PROTEIN_TYROSINE_PHOSPHATASE_ACTIVITY (P = 5.24E−3), and down-regulated miRNAs were associated with the term POSITIVE_REGULATION_OF_JNK_ACTIVITY (P 9.47e−4). Predicted target genes associated with phosphatase activity and dephosphorylation included MTMR3, PTPN18, DUSP5, PTPN2, DUSP2, and PPP1CA.

### MiRNA Expression in Liver Biopsy Samples

We compared miRNA expression in non-cancerous liver biopsy samples from a patient with chronic HBV to two uninfected patients (Table S2, Fig. S8). MiRNA levels were highly correlated between liver tissue and serum in all patients (P = <0.001; R^2^ = 0.57), including the top HBV-associated miRNAs identified by microarray and RT-PCR analysis in this study.

### Co-localization of HBcAg and HBsAg with AGO2

Using immunocytochemistry and PLA analysis, we found that HBV core protein and AGO2 co-localized within T23 cells (Fig. 1A–B), suggesting a potential protein-protein interaction between HBcAg and AGO2. AGO2 also co-localized with HBs in T23 cells (Fig. 1C), indicating a potential interaction between HBs and AGO2. Overlap between anti-HBc and anti-HBs staining (Fig. 1D) and between anti-AGO2, anti-HBc, and anti-HBs (Fig. 1E) suggests that these three proteins may co-localize. No overlap was observed between anti-AGO2 and anti-HBx staining in HepG2 cells transfected with HBx expression plasmid (p3FLAG-HBx) nor in control cells, suggesting that HBx does not interact with AGO2 (data not shown).

### Subcellular Localization

We also examined HBcAg sub-cellular localization using immunocytochemistry and PLA analysis and found that HBcAg localized to several intracellular compartments, including the ER, autophagosomes, endosomes, and Golgi (Fig. 2). No evidence was found for interaction with mitochondria (data not shown). Using immunocytochemistry, HBsAg was also found to localize diffusely to several intracellular compartments, including the ER, endosomes, autophagosomes, Golgi, mitochondria, processing bodies, multi-vesicular bodies, and the nuclear envelope (Fig. 3). HBx localized non-specifically in the nucleus and cytoplasm, and no sub-cellular location could be ascertained (Fig. S9).

### RNA Interference against AGO2

Antisense RNA directed against AGO2 strongly suppressed AGO2 expression (Fig. 4A) and resulted in lower HBV DNA (Fig. 4B) and HBsAg (Fig. 4C) levels in the supernatant. Cell viability was not significantly reduced (Fig. 4D).

## Discussion

In this study, we report a set of miRNAs that were up-regulated in serum of HBV infected individuals compared to healthy controls. Mir-122, miR-22, miR-99a, and miR-125b in particular, were significantly elevated in serum of HBV patients. We also showed that AGO2, an essential component of the RNA silencing complex, co-localizes with both HBc and HBs proteins. HBc and/or HBs localize to several organelles associated with protein synthesis, processing, and degradation, including the ER, Golgi, endosomes, autophagosomes, processing bodies, and multivesicular bodies. Although we expected that depletion of AGO2 would relieve inhibition of HBV replication, we found instead that knockdown of AGO2 appears to inhibit HBV replication, implying that HBV may require AGO2 during its life cycle.

The role of AGO2 is unclear, but viruses have previously been shown to interfere with elements of the RNA-induced gene silencing pathway [17]. HCV core protein and the HIV-1 Tat protein suppress gene silencing by inhibiting Dicer, a cytoplasmic protein that processes pre-microRNA [18]. HBV down-regulates expression of Drosha, the nuclear protein involved in the first step of miRNA processing, which might globally suppress miRNA expression levels [19]. Viruses also influence expression of individual miRNAs [17].

Considering that miR-122 strongly suppresses HBV replication, it is curious that HBV is nonetheless often able to establish chronic infection in the liver [20], [21], [22]. In the case of HCV, miR-122/AGO2 binding stabilizes the HCV genome and prevents degradation, such that suppression of either miR-122 or AGO2 inhibits HCV replication [23], [24], [25]. In HBV, we also found that AGO2 knockdown suppresses replication, but Wang et al. demonstrated that anti-sense depletion of miR-122 promoted HBV replication instead of suppressing it [26]. MiR-122 suppresses HBV replication both through direct binding to HBV RNA as well as indirectly through cyclin G1-modulated p53 activity [20], [27], [28]. HBV might therefore be expected to down-regulate miR-122 levels to evade miR-122 binding and suppression. Wang et al. indeed found that miR-122 levels are significantly decreased in the liver of chronic HBV patient [26], whereas elevated miR-122 levels in the serum have been reported [4], [29].

One explanation for the discrepancy between liver and serum miR-122 levels might be that HBV sequesters and expels AGO2-bound miR-122 inside of HBsAg particles, possibly along with other miRNAs that interfere with the viral life cycle. HBV vastly over-produces surface proteins that self-assemble into what were initially thought to be empty particles [30], [31], but which may contain miRNAs stably bound to AGO2 [5]. Although HBV is a DNA virus, it relies on reverse transcription via an RNA intermediate in a way similar to retroviruses. Bouttier et al. showed that two unrelated retroviruses, HIV-1 and PFV-1, both require AGO2 interaction with viral RNA for assembly of viral particles. In these viruses, AGO2 is recruited to viral RNA and encapsidated along with it without impairing translation of viral RNA [32]. This suggests that some viruses may take advantage of another function of Argonaute, such as its role in the formation of P-bodies [33], although AGO2 possesses intrinsic exonuclease activity that must be countered. AGO2-mediated gene silencing requires recruitment of GW182 via multiple GW-rich regions [34]. While HIV-1 and PFV-1 encapsidate AGO2, they do not encapsidate GW182, which might provide a means to suppress AGO2 silencing. Some plant viruses use molecular mimicry to inhibit RISC activity by binding to Argonaute proteins through virally encoded WG/GW motifs [35]. Although HBV proteins appear to lack WG/GW motifs, the HBV core protein may use a similar mechanism to disrupt RISC activity while preserving other AGO2 functions. One possibility involves HSP90, a chaperone involved in maintenance of the polymerase/pgRNA complex. HSP90 binds to HBV core protein dimers and is internalized in capsids, but it also binds to the N-terminus of AGO2 and may be required for miRNA loading and targeting to P-bodies [36], [37]. Co-localization studies with other proteins and analysis of bound miRNAs may be necessary to elucidate the role of AGO2 in HBV replication, but we speculate that HBV proteins might suppress miRNA activity by binding to and sequestering AGO2 and their bound miRNAs.

Pathway analysis of the predicted targets of the up-regulated serum miRNAs in HBV patients showed that genes involved in phosphatase activity were significantly over-represented. Each of several miRNAs, including miR-122, miR-125b, and miR-99a, was predicted to target a different phosphorylation-associated gene. Regulation of phosphorylation appears to be important in HBV replication, as phosphorylation of the C terminal domain of the HBV core protein is essential for pgRNA packaging and HBV capsid maturation [38]. Phosphorylation also inhibits AGO2 binding of miRNA [39] and is involved in localization to P-bodies [40]. Recent studies have demonstrated that HBV enhances and exploits autophagy via the HBx and small HBs proteins to promote viral DNA replication and envelopment without increasing the rate of protein degradation [41], [42]. Sir et al suggested that autophagy may affect dephosphorylation and maturation of the core protein, which protects viral DNA during replication [43]. These reports suggest that HBV exploits multiple cellular pathways in order to establish an intracellular environment conducive to replication.

Although many HBV-associated miRNAs have been reported, the functions of only a few have been examined. MiR-122, miR-125a-5p, miR-199a-3p and miRNA-210 have all been reported to bind to and directly suppress HBV RNA [8], [27], [44], whereas other miRNAs have been shown to promote or suppress HBV replication indirectly. MiR-1 enhances HBV core promoter activity by up-regulating FXRα, a transcription factor essential for HBV replication [45], whereas miR-141 suppresses HBsAg production in HepG2 cells by down-regulating promoter activity via PPARA [46]. The role of miR-22 and miR-99a in HBV infection is less clear, but both are involved in regulation of cell fate and are implicated in development of HCC. MiR-99a is one of the most highly expressed miRNAs in normal liver tissue and is severely down-regulated in HCC and other cancers, suggesting a role as a tumor suppressor [47]. MiR-99a alters sensitivity to TGF-β activity by suppressing phosphorylation of SMAD3 [48], whereas the HBx protein disrupts TGF-β signaling by shifting from the pSmad3C pathway to the oncogenic pSmad3L pathway [49]. MiR-22 acts as a tumor suppressor by inducing cellular senescence and is down-regulated in several cancer lines [50]. However, over-expression of miR-22 in males is associated with down-regulation of ERα expression, which compromises the protective effect of estrogen and leads to up-regulation of IL-1α in hepatocytes under stress caused by reactive oxygen species, which is another hallmark of HBx interference [51]. Differences in miRNA levels between hepatic and serum miRNA profiles may reveal miRNAs that play an essential role in the HBV life cycle, with potential application to miRNA-based diagnosis and therapy.

In this study we demonstrated potential interactions between AGO2 and HBc and HBs, but not HBx, in stably transfected HepG2 cells. Suppression of HBV DNA and HBsAg in the supernatant following AGO2 knockdown and the presence of HBV-associated miRNAs in the serum may indicate a dependency on AGO2 during the HBV life cycle.

## Supporting Information

Figure S1 **Heat map of miRNA expression.** Healthy controls and patients with chronic HBV clustered separately based on serum miRNA expression. “Healthy males” and “healthy females” refer to serum mixtures of 12 uninfected males and 10 uninfected females, respectively. “HBV low” and “HBV high” refer to serum mixtures from 10 patients with low (≤42 IU/l) ALT levels and 10 patients with high ALT levels (>42 IU/l), respectively.
(TIF)

Figure S2 **Pairwise correlations among pooled serum miRNA samples.** Pooled serum samples were collected from 10 healthy males, 10 healthy females, 10 HBV patients with low ALT levels, and 10 HBV patients with high ALT levels. Pairwise correlations in miRNA expression levels among all four pooled samples were strong (>0.90; P<0.001), but correlations were strongest between the healthy male and female samples (0.98) and between the low and high ALT HBV patients (0.98), suggesting that expression of a subset of miRNAs is altered during HBV infection.
(TIF)

Figure S3 **Relationship between serum miRNAs and HBsAg levels in chronic HBV patients.** Serum levels of several miRNAs were significantly correlated with HBsAg levels in patients with chronic HBV. MiR-99a, miR-122, and miR-125b levels were most strongly correlated with HBsAg levels, with R^2^ of 0.69, 0.56, and 0.54, respectively.
(TIF)

Figure S4 **Relationship between serum miRNAs and HBV DNA levels in chronic HBV patients.** Serum levels of several miRNAs were significantly correlated with HBV DNA levels in patients with chronic HBV. MiR-122, miR-99a, and miR-125b levels were most strongly correlated with HBV DNA levels, with R^2^ of 0.44, 0.43, and 0.39, respectively.
(TIF)

Figure S5 **Relationship between serum miRNAs and ALT levels in chronic HBV patients.** Serum levels of several miRNAs were significantly but somewhat diffusely correlated with ALT levels in patients with chronic HBV. MiR-122 and miR-22 levels were correlated with ALT levels with R^2^ of 0.25 and 0.21, respectively.
(TIF)

Figure S6 **Relationship between serum miRNAs and presence of HBe antigen in chronic HBV patients.** Serum levels of miR-122, miR-99a, miR-720, and miR-125b were significantly elevated in patients positive for the HBe antigen.
(TIF)

Figure S7 **Relationship between serum miRNAs and presence of HBe antibody in chronic HBV patients.** Serum levels of miR-122, miR-99a, miR-720, and miR-125b were significantly elevated in patients negative for the HBe antibody.
(TIF)

Figure S8 **Relationship between individual miRNAs in the liver and serum.** Each point represents the level of a specific miRNA in non-cancerous liver tissue relative to serum in the same patient. Red points represent miRNA levels from a patient with chronic HBV, and blue and green points correspond to two different uninfected control subjects. Large red points and labels indicate the subset of miRNAs (Tables 2 and 3) that were significantly elevated in serum of chronic HBV patients. MiRNA expression levels were positively correlated (R^2^ = 0.57; P<2.1E-16) between liver tissue and serum, suggesting that serum levels broadly reflect miRNA levels in the liver. There appears to be no clear discrepancy between liver and serum miRNA levels in the HBV-infected patient compared to the two uninfected patients.
(TIF)

Figure S9 **Subcellular localization of HBx analyzed by immunocytochemistry.** HBx localized non-specifically in the nucleus and cytoplasm, but we were unable to verify the sub-cellular location. Anti-Rab5 staining for endosomes is shown for illustration, but results were similar using antibodies against other compartments.
(TIF)

Table S1 **Antibodies used for immunocytochemistry.**
(DOC)

Table S2 **Significantly up- or down-regulated miRNAs in liver samples from an HBV-infected patient compared to two non-HBV-infected patients.**
(DOC)
