# Supplementary figures and images for: Hepatitis B Virus-Specific miRNAs and Argonaute2 Play a Role in the Viral Life Cycle
Source: PLoS One. 2012 Oct 16;7(10):e47490. doi: 10.1371/journal.pone.0047490 (PMC3472984; doi:10.1371/journal.pone.0047490)

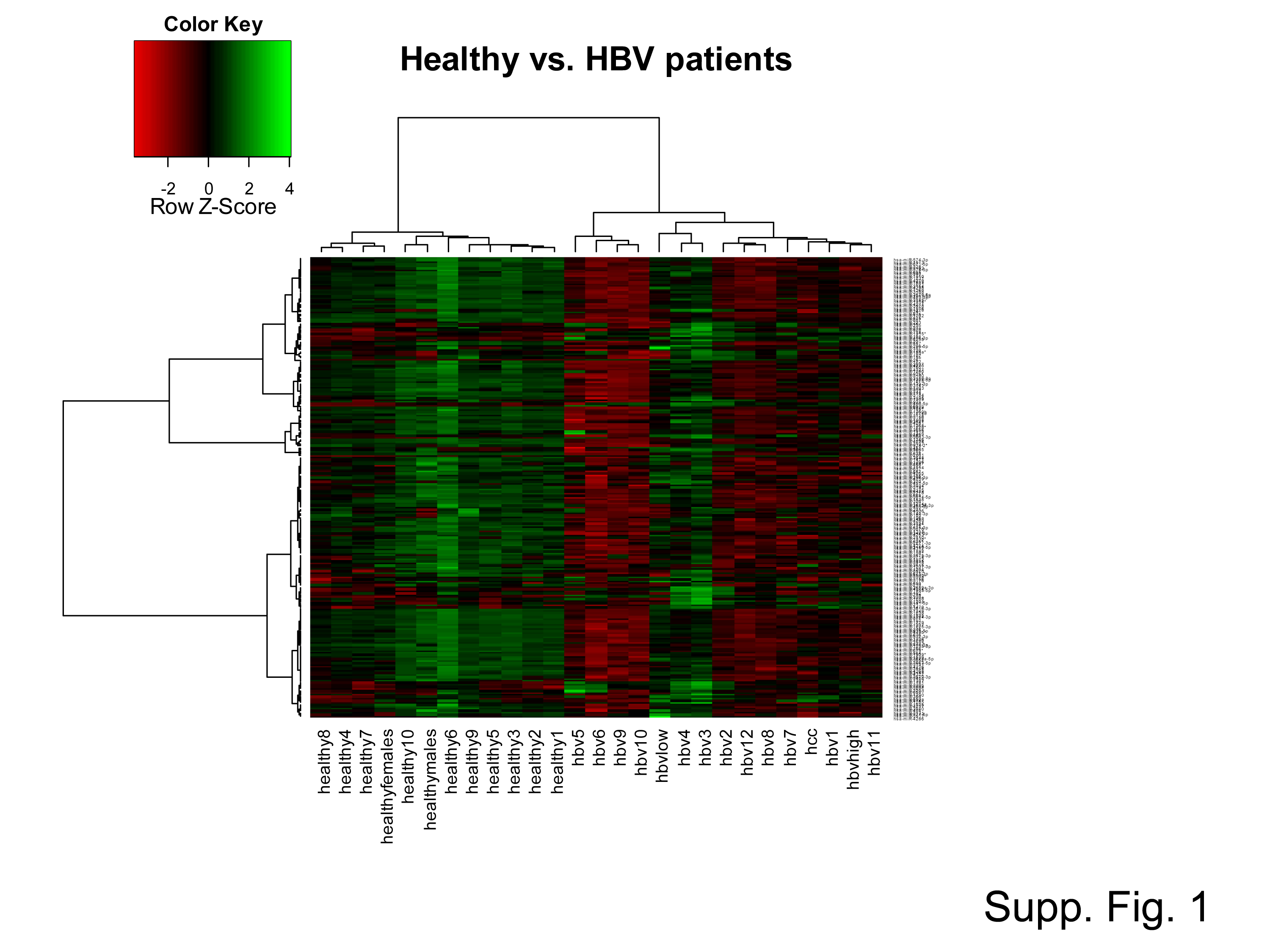

Supplement: Figure S1 — Heat map of miRNA expression. Healthy controls and patients with chronic HBV clustered separately based on serum miRNA expression. “Healthy males” and “healthy females” refer to serum mixtures of 12 uninfected males and 10 uninfected females, respectively. “HBV low” and “HBV high” refer to serum mixtures from 10 patients with low (≤42 IU/l) ALT levels and 10 patients with high ALT levels (>42 IU/l), respectively. (TIF) [file pone.0047490.s001.tif]

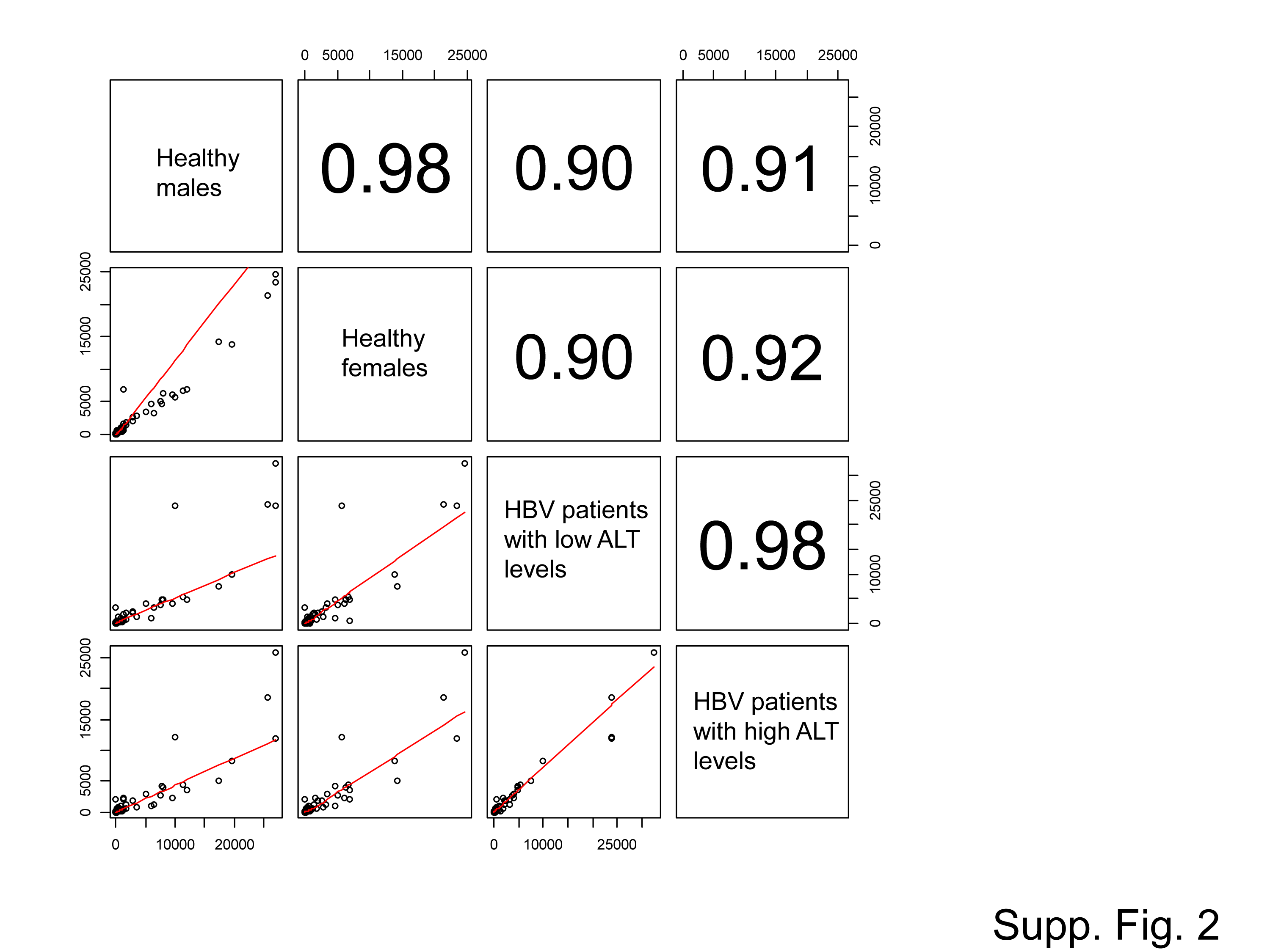

Supplement: Figure S2 — Pairwise correlations among pooled serum miRNA samples. Pooled serum samples were collected from 10 healthy males, 10 healthy females, 10 HBV patients with low ALT levels, and 10 HBV patients with high ALT levels. Pairwise correlations in miRNA expression levels among all four pooled samples were strong (>0.90; P<0.001), but correlations were strongest between the healthy male and female samples (0.98) and between the low and high ALT HBV patients (0.98), suggesting that expression of a subset of miRNAs is altered during HBV infection. (TIF) [file pone.0047490.s002.tif]

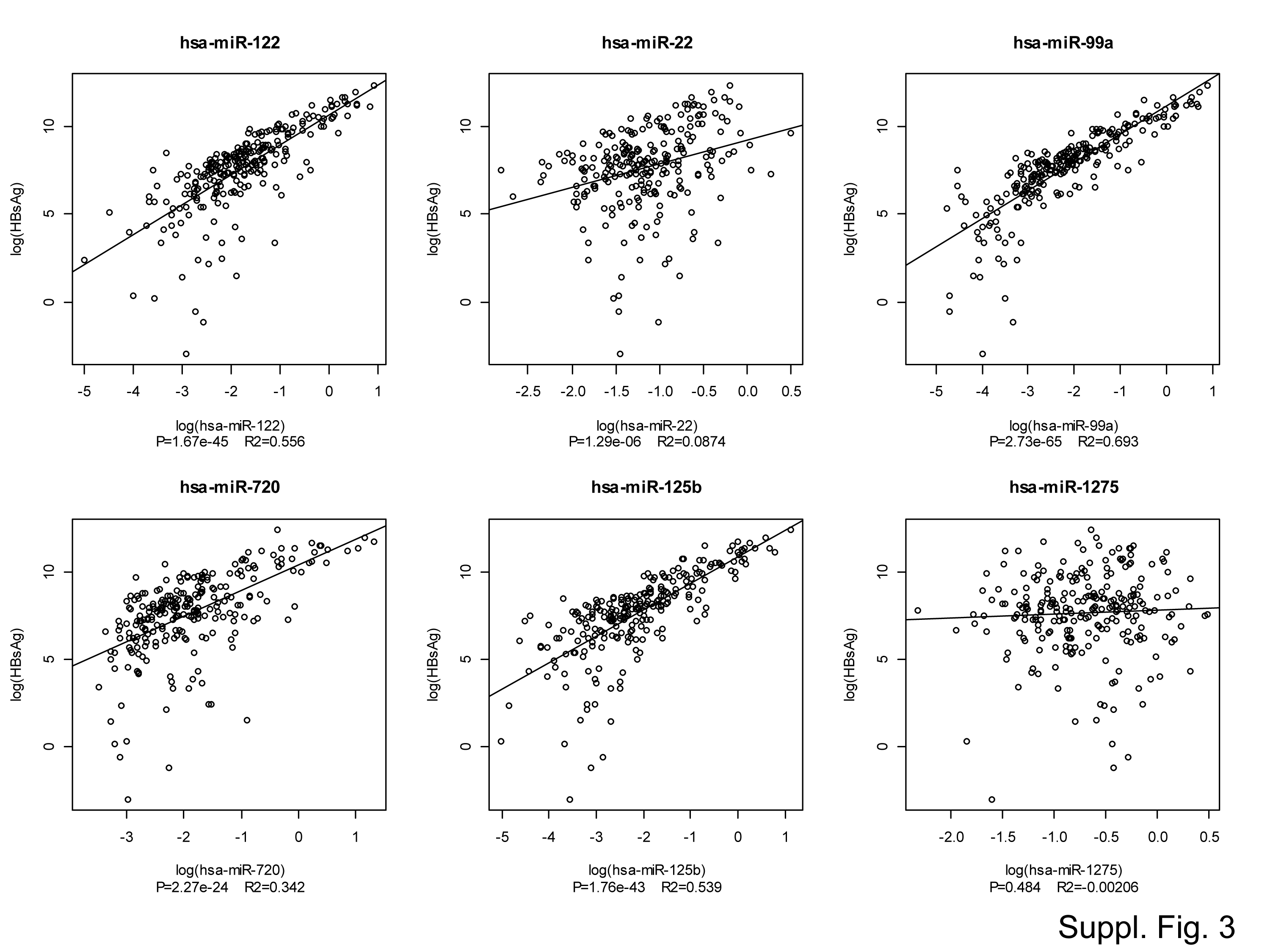

Supplement: Figure S3 — Relationship between serum miRNAs and HBsAg levels in chronic HBV patients. Serum levels of several miRNAs were significantly correlated with HBsAg levels in patients with chronic HBV. MiR-99a, miR-122, and miR-125b levels were most strongly correlated with HBsAg levels, with R2 of 0.69, 0.56, and 0.54, respectively. (TIF) [file pone.0047490.s003.tif]

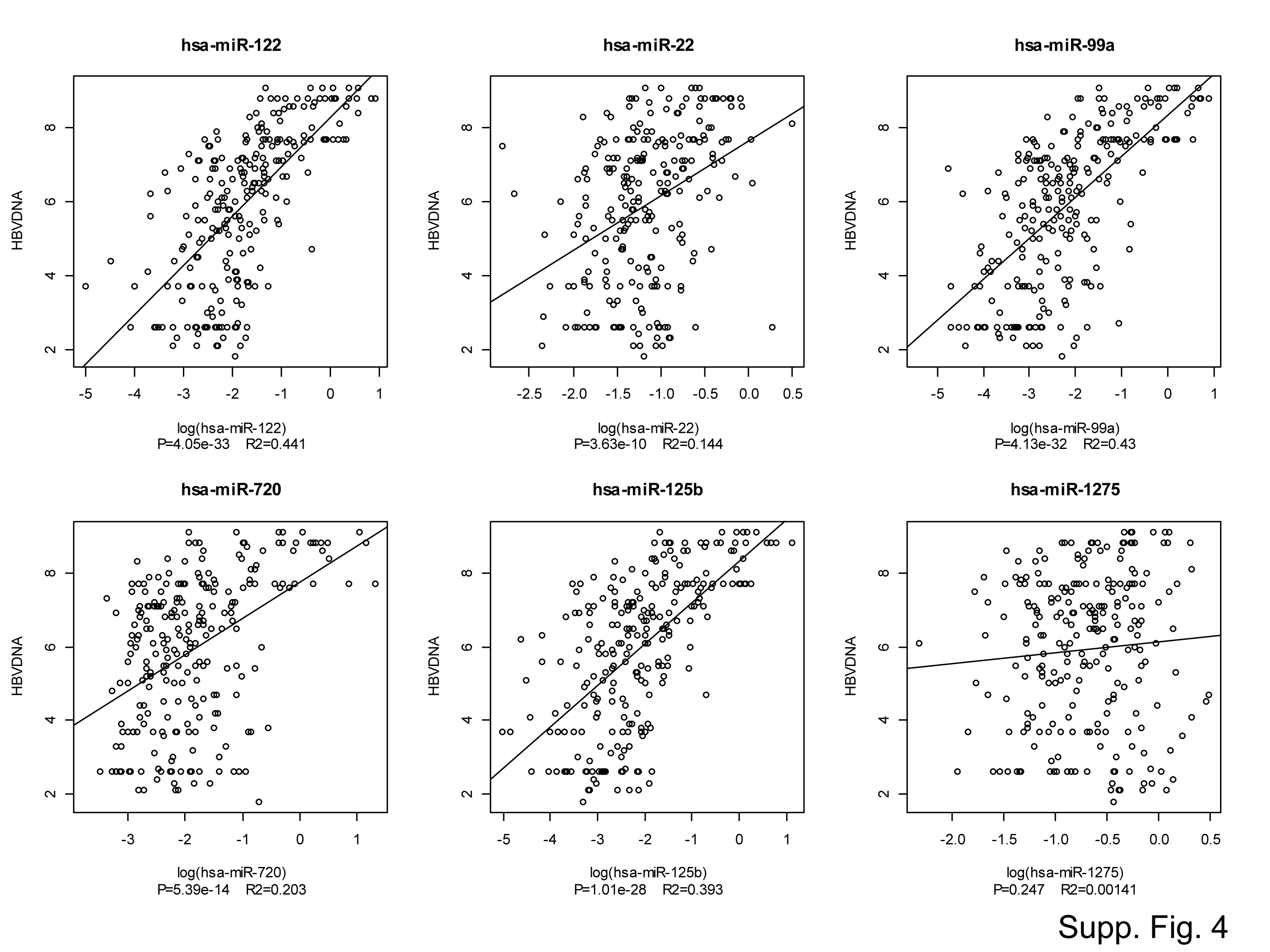

Supplement: Figure S4 — Relationship between serum miRNAs and HBV DNA levels in chronic HBV patients. Serum levels of several miRNAs were significantly correlated with HBV DNA levels in patients with chronic HBV. MiR-122, miR-99a, and miR-125b levels were most strongly correlated with HBV DNA levels, with R2 of 0.44, 0.43, and 0.39, respectively. (TIF) [file pone.0047490.s004.tif]

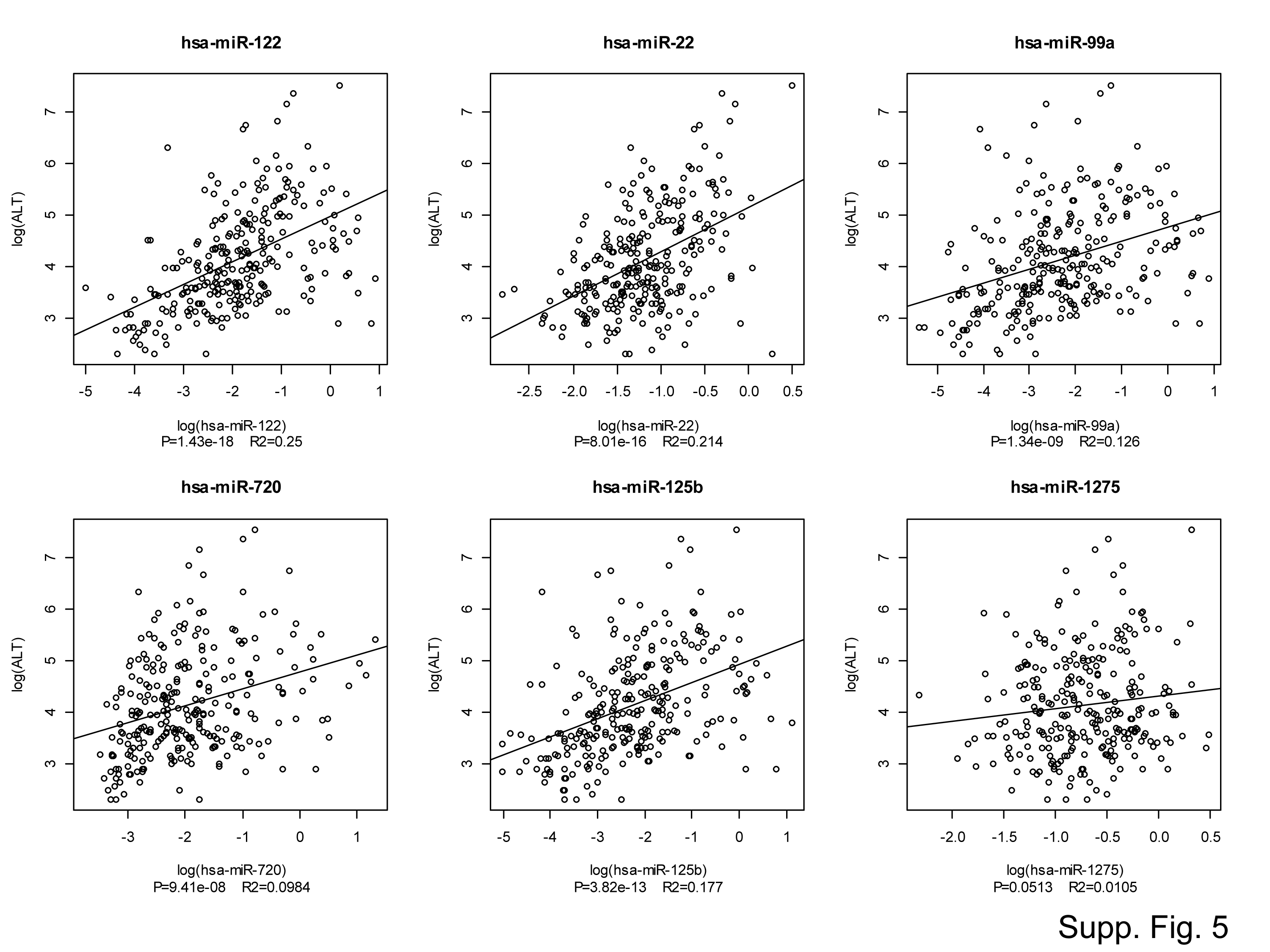

Supplement: Figure S5 — Relationship between serum miRNAs and ALT levels in chronic HBV patients. Serum levels of several miRNAs were significantly but somewhat diffusely correlated with ALT levels in patients with chronic HBV. MiR-122 and miR-22 levels were correlated with ALT levels with R2 of 0.25 and 0.21, respectively. (TIF) [file pone.0047490.s005.tif]

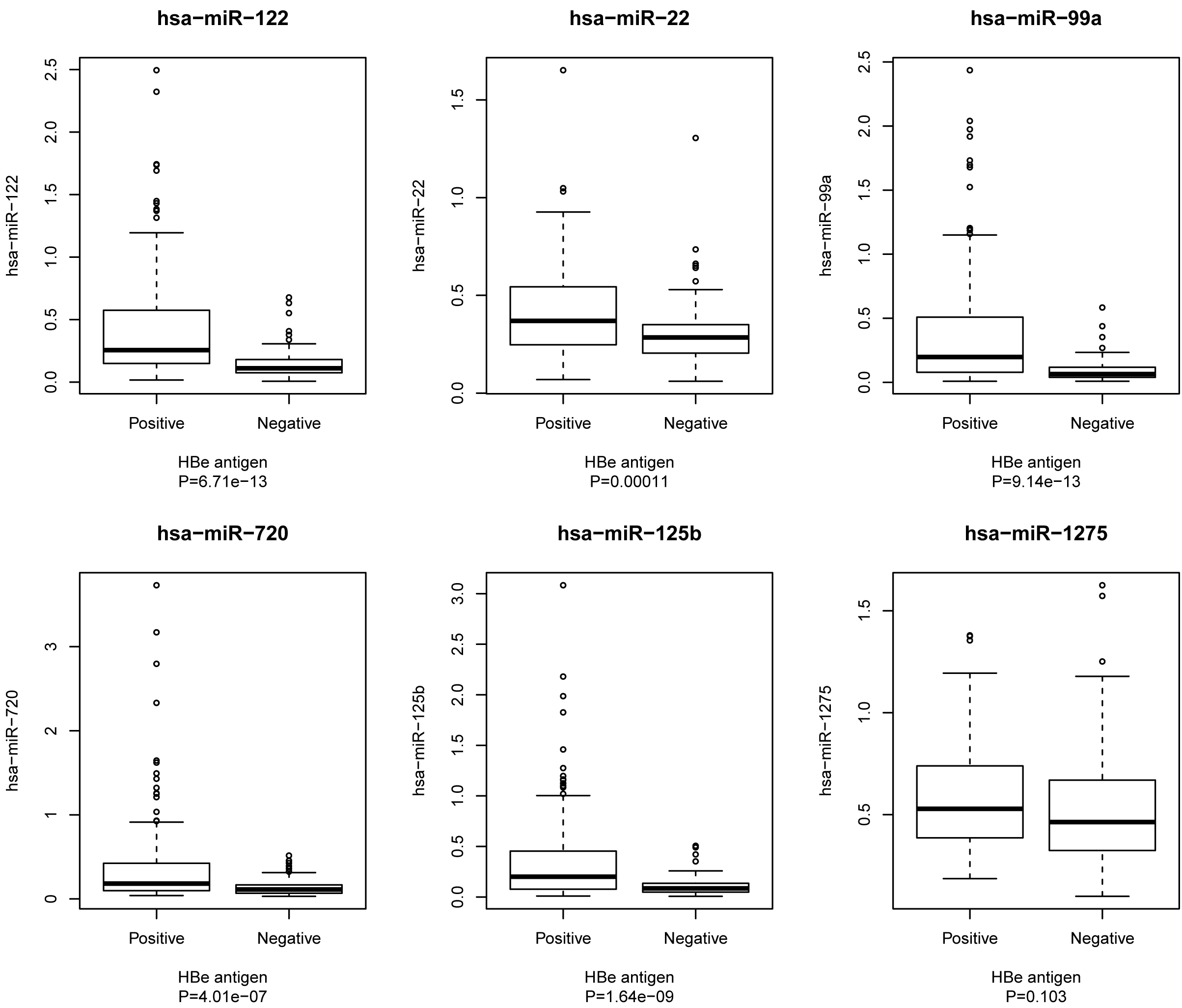

Supplement: Figure S6 — Relationship between serum miRNAs and presence of HBe antigen in chronic HBV patients. Serum levels of miR-122, miR-99a, miR-720, and miR-125b were significantly elevated in patients positive for the HBe antigen. (TIF) [file pone.0047490.s006.tif]

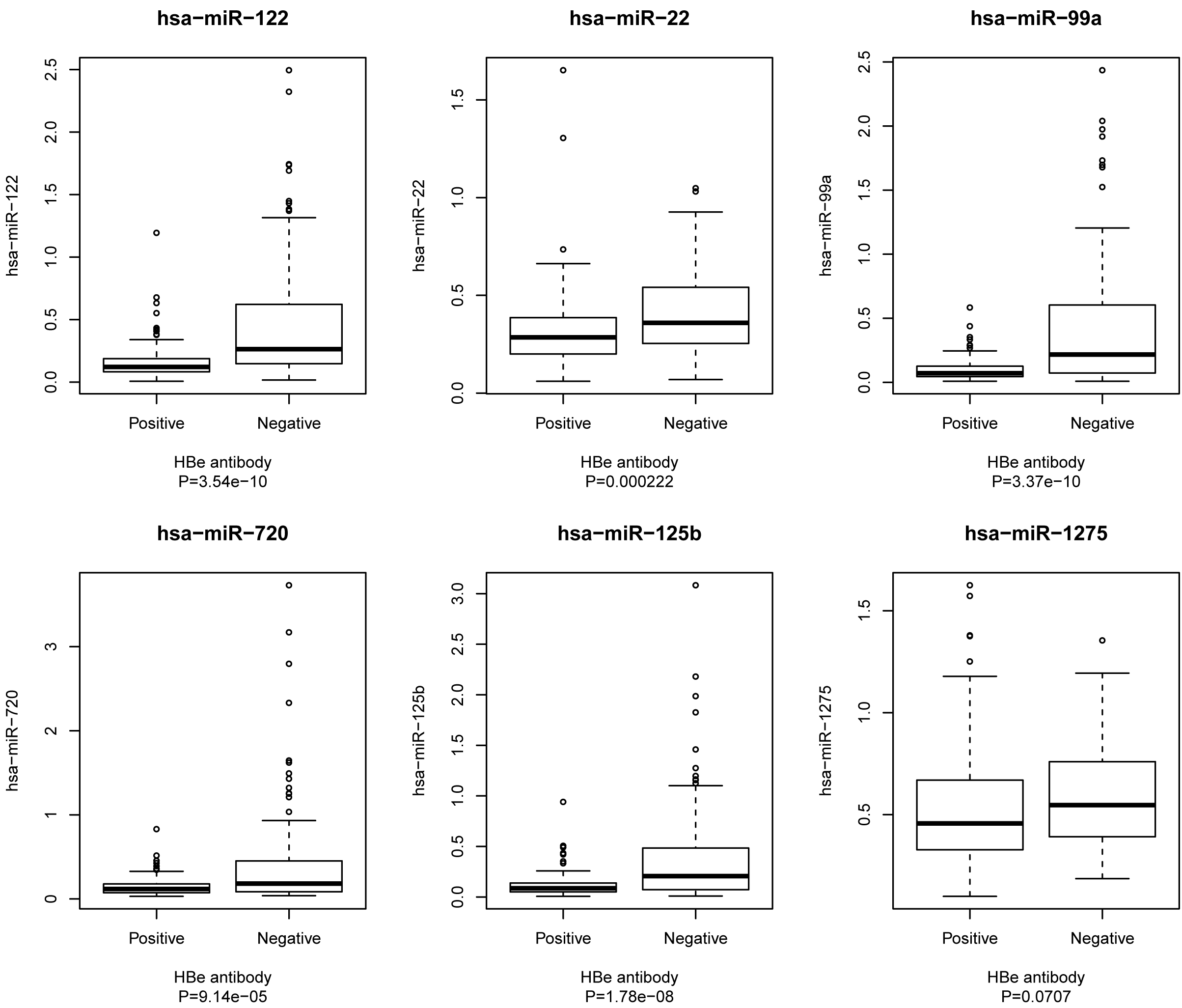

Supplement: Figure S7 — Relationship between serum miRNAs and presence of HBe antibody in chronic HBV patients. Serum levels of miR-122, miR-99a, miR-720, and miR-125b were significantly elevated in patients negative for the HBe antibody. (TIF) [file pone.0047490.s007.tif]

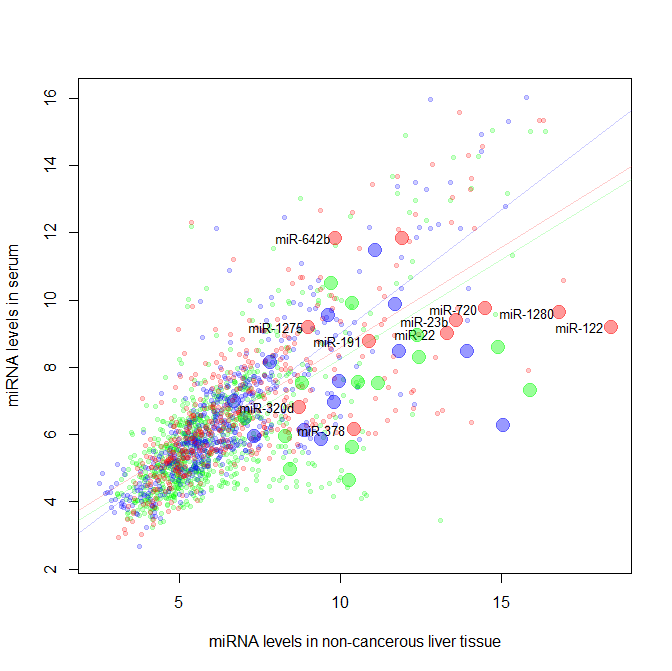

Supplement: Figure S8 — Relationship between individual miRNAs in the liver and serum. Each point represents the level of a specific miRNA in non-cancerous liver tissue relative to serum in the same patient. Red points represent miRNA levels from a patient with chronic HBV, and blue and green points correspond to two different uninfected control subjects. Large red points and labels indicate the subset of miRNAs (Tables 2 and 3) that were significantly elevated in serum of chronic HBV patients. MiRNA expression levels were positively correlated (R2 = 0.57; P<2.1E-16) between liver tissue and serum, suggesting that serum levels broadly reflect miRNA levels in the liver. There appears to be no clear discrepancy between liver and serum miRNA levels in the HBV-infected patient compared to the two uninfected patients. (TIF) [file pone.0047490.s008.tif]

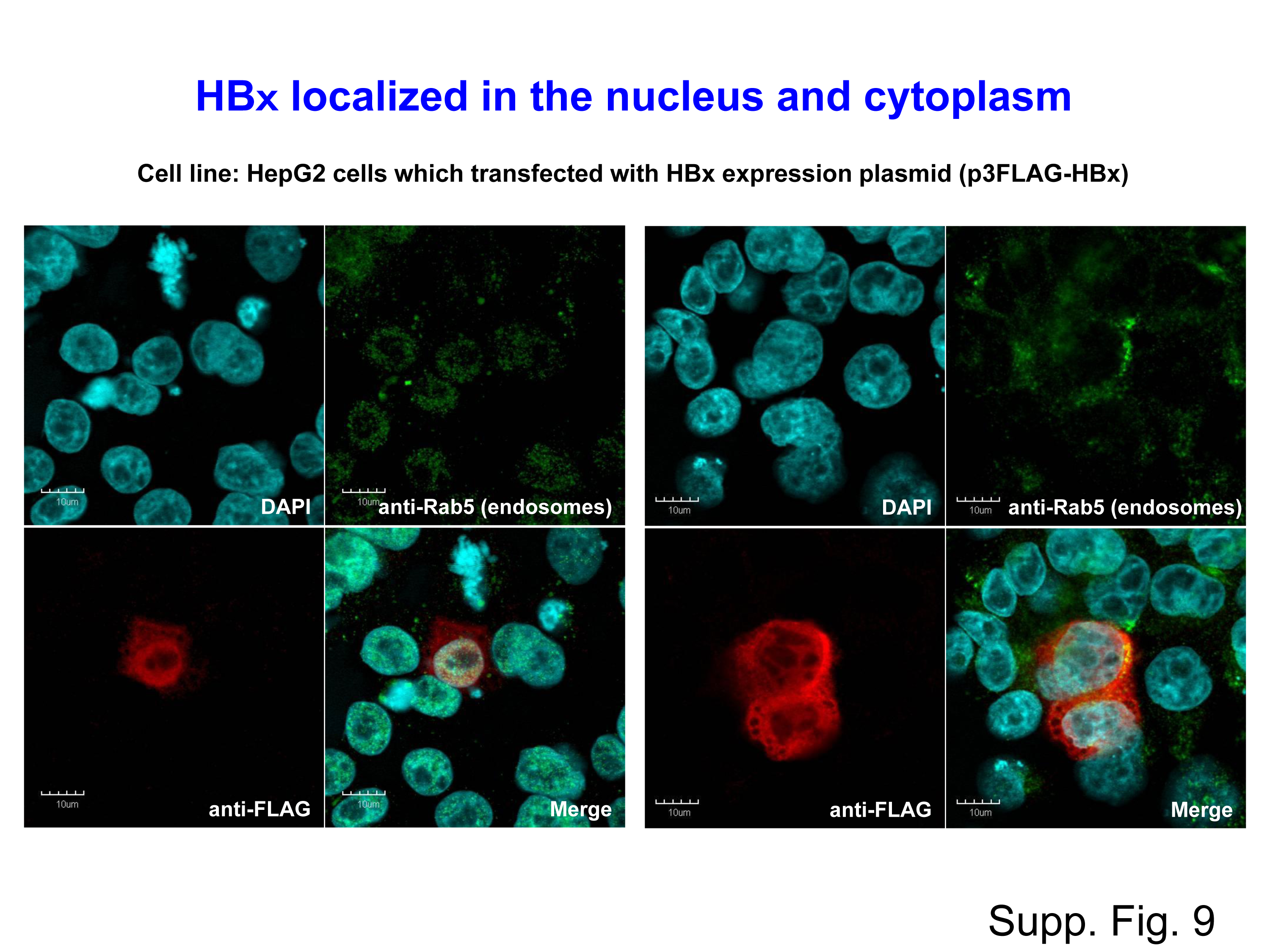

Supplement: Figure S9 — Subcellular localization of HBx analyzed by immunocytochemistry. HBx localized non-specifically in the nucleus and cytoplasm, but we were unable to verify the sub-cellular location. Anti-Rab5 staining for endosomes is shown for illustration, but results were similar using antibodies against other compartments. (TIF) [file pone.0047490.s009.tif]
