# Supplementary material for: Hepatitis B Virus-Specific miRNAs and Argonaute2 Play a Role in the Viral Life Cycle
Source: PLoS One. 2012 Oct 16;7(10):e47490. doi: 10.1371/journal.pone.0047490 (PMC3472984; doi:10.1371/journal.pone.0047490)
Supplement: Table S1 — Antibodies used for immunocytochemistry. (DOC) [file pone.0047490.s010.doc]

Table S1. Antibodies used for immunocytochemistry.

| Target protein | Antibody | Dilution |
| --- | --- | --- |
| HBV core protein (HBc) | Mouse monoclonal antibody against hepatitis B core antigen (Clone No. 3105, Institute of Immunology, Tokyo, Japan) | 1:500 |
| HBV surface antigen (HBs) | Mouse monoclonal antibody against hepatitis B surface antigen (Hyb-5124A, Institute of Immunology, Tokyo, Japan) | 1:500 |
| HBV X protein (HBx) | Anti-FLAG M2 monoclonal antibody (SIGMA, Saint Louis, Missouri, USA) | 1:500 |
| Argonaute 2 (AGO2) | Rabbit monoclonal antibody against Argonaute 2 (C34C6, Cell signaling Technology Japan, Tokyo, Japan) | 1:500 |
| Calnexin (Endoplasmic reticulum) | Rabbit monoclonal antibody against Calnexin (C5C9, Cell signaling Technology Japan, Tokyo, Japan) | 1:500 |
| COX IV (Mitochondria) | Rabbit monoclonal antibody against COX IV (3E11, Cell signaling Technology Japan, Tokyo, Japan) | 1:500 |
| Rab5 (Endosome) | Rabbit monoclonal antibody against Rab5 (C8B1, Cell signaling Technology Japan, Tokyo, Japan) | 1:500 |
| LC3B (Autophagosome) | Rabbit monoclonal antibody against LC3B (D11, Cell signaling Technology Japan, Tokyo, Japan) | 1:500 |
| Golgi protein | Rabbit polyclonal antibody against 58K Golgi protein (ab5820, abcam, Tokyo, Japan) | 1:500 |
| EDC4/Ge-1 (Processing body) | Rabbit monoclonal antibody against EDC4/Ge-1 (Cell signaling Technology Japan, Tokyo, Japan) | 1:500 |
| CHMP4B (MVB) | Rabbit monoclonal antibody against CHMP4B (Abcam, Cambridge, UK) | 1:500 |
