# Supplementary material for: Hepatitis B Virus-Specific miRNAs and Argonaute2 Play a Role in the Viral Life Cycle
Source: PLoS One. 2012 Oct 16;7(10):e47490. doi: 10.1371/journal.pone.0047490 (PMC3472984; doi:10.1371/journal.pone.0047490)
Supplement: Table S2 — Significantly up- or down-regulated miRNAs in liver samples from an HBV-infected patient compared to two non-HBV-infected patients. (DOC) [file pone.0047490.s011.doc]

Table S2. Significantly up- or down-regulated miRNAs in liver samples from an HBV-infected patient compared to two non-HBV-infected patients.

| Direction | miRNA | logFC | AveExpr | t | P | P_FDR_ |
| --- | --- | --- | --- | --- | --- | --- |
| Up | hsa-miR-4284 | 3.85 | 11.52 | 7.98 | 0.000101 | 0.047 |
|  | hsa-miR-4286 | 3.79 | 13.33 | 8.63 | 6.13E-05 | 0.047 |
|  | hsa-miR-23b | 3.11 | 11.54 | 4.99 | 0.00165 | 0.047 |
|  | hsa-let-7f | 3.09 | 9.51 | 5.32 | 0.00116 | 0.047 |
|  | hsa-miR-22 | 3.08 | 11.29 | 6.61 | 0.00032 | 0.047 |
|  | hsa-let-7g | 3.06 | 9.25 | 6.13 | 0.00051 | 0.047 |
|  | hsa-miR-122 | 2.93 | 16.47 | 5.85 | 0.00067 | 0.047 |
|  | hsa-miR-194 | 2.89 | 11.52 | 6.31 | 0.00043 | 0.047 |
|  | hsa-miR-30b | 2.85 | 9.90 | 6.42 | 0.00038 | 0.047 |
|  | hsa-miR-192 | 2.77 | 11.34 | 6.05 | 0.00055 | 0.047 |
| Down | hsa-miR-3687 | -3.23 | 8.64 | -6.87 | 0.00025 | 0.047 |
|  | hsa-miR-708 | -2.36 | 6.02 | -4.98 | 0.00167 | 0.047 |

logFC: log2 fold-change between patients with chronic HBV infection relative to healthy individuals.

AveExpr: The average log2 expression level for each miRNA over all samples.

t: moderated t-statistic for patients with chronic HBV infection compared to healthy individuals P for each miRNA

P: uncorrected P-value for t-test.

P_FDR_: P-value adjusted for multiple testing based on the false discovery rate.
